# Understanding heterogeneous tumor microenvironment in metastatic melanoma

**DOI:** 10.1371/journal.pone.0216485

**Published:** 2019-06-05

**Authors:** Yiyi Yan, Alexey A. Leontovich, Michael J. Gerdes, Keyur Desai, Jinhong Dong, Anup Sood, Alberto Santamaria-Pang, Aaron S. Mansfield, Chrystal Chadwick, Rong Zhang, Wendy K. Nevala, Thomas J. Flotte, Fiona Ginty, Svetomir N. Markovic

**Affiliations:** 1 Division of Medical Oncology, Mayo Clinic, Rochester, Minnesota, United States of America; 2 Division of Biomedical Statistics and Informatics, Mayo Clinic, Rochester, Minnesota, United States of America; 3 Diagnostics, Imaging and Biomedical Technologies, GE Global Research Center, Niskayuna, New York, United States of America; 4 Clinical Immunology and Immunotherapeutics, Mayo Clinic, Rochester, Minnesota, United States of America; 5 Division of Hematology, Mayo Clinic, Rochester, Minnesota, United States of America; 6 Division of Anatomic Pathology and Division of Dermatopathology and Cutaneous Immunopathology, Mayo Clinic, Rochester, Minnesota, United States of America; Istituto Superiore di Sanità, ITALY

## Abstract

A systemic analysis of the tumor-immune interactions within the heterogeneous tumor microenvironment is of particular importance for understanding the antitumor immune response. We used multiplexed immunofluorescence to elucidate cellular spatial interactions and T-cell infiltrations in metastatic melanoma tumor microenvironment. We developed two novel computational approaches that enable infiltration clustering and single cell analysis—cell aggregate algorithm and cell neighborhood analysis algorithm—to reveal and to compare the spatial distribution of various immune cells relative to tumor cell in sub-anatomic tumor microenvironment areas. We showed that the heterogeneous tumor human leukocyte antigen-1 expressions differently affect the magnitude of cytotoxic T-cell infiltration and the distributions of CD20+ B cells and CD4+FOXP3+ regulatory T cells within and outside of T-cell infiltrated tumor areas. In a cohort of 166 stage III melanoma samples, high tumor human leukocyte antigen-1 expression is required but not sufficient for high T-cell infiltration, with significantly improved overall survival. Our results demonstrate that tumor cells with heterogeneous properties are associated with differential but predictable distributions of immune cells within heterogeneous tumor microenvironment with various biological features and impacts on clinical outcomes. It establishes tools necessary for systematic analysis of the tumor microenvironment, allowing the elucidation of the “homogeneous patterns” within the heterogeneous tumor microenvironment.

## Introduction

The success of cancer immune checkpoint therapies depends on their ability to mobilize immune cells and direct their activities into the highly heterogeneous tumor microenvironment (TME), where they exert their antitumor effects. However, even with the use of modern day immunotherapeutics, durable clinical responses are achieved in only a subset of patients with advanced cancer [1]. Most cancer immunotherapy efforts, including immune checkpoint inhibitors (e.g., anti–programmed cell death protein 1 [PD1] antibodies), are aimed at maximizing the effect of tumor-infiltrating lymphocytes (TILs) through different approaches [2–5]. Presence of TILs within the TME correlates with clinical benefit in multiple malignancies [6–9]. Physical proximity and contact between immune (cytotoxic T lymphocytes) and tumor cells within the TME is essential for effective antitumor immunotherapy. The variable levels of immune cell tumor infiltration and the tumor human leukocyte antigen class I antigens (HLA-1) and PD-L1 expressions have been attributed to the variable clinical outcomes [10]. The TME is a heterogeneous mixture of various immune and tumor cells with different functional phenotypes and spatial distribution patterns. The outcome of dynamic interactions between cells with different functional profiles within micro-anatomic locations of TME will ultimately determine the success of immune destruction of tumors, collectively determining the outcome of immunotherapy. Therefore, understanding the functional and spatial interplays between tumor and different immune populations will likely provide insight to the variable treatment responses and to improve outcomes of cancer immunotherapy. However, due to the complexity of the TME heterogeneity, tools for systematic analysis of the micro-anatomic in situ cellular level interplay are currently lacking.

In recent years, there has been a broad effort in technology development to address this challenge. To overcome this barrier in understanding the tumor-associated immune response, strategies have evolved to utilize clinically processed formalin fixed and paraffin embedded (FFPE) human tumor tissues in a highly multiplexed manner to provide a comprehensive picture of multiparametrically defined individual cells and their relative anatomic locations in situ, using surgical specimens [11–13]. These strategies all provide an enormous amount of data on cells and cell-level marker expression; tools are just beginning to emerge that take into context the spatial location of different cells and how they relate to and interact with one another.

In this study, we used Cell DIVE technology (GE Healthcare) [14, 15] in combination with novel spatial analytical tools adapted from the ecology field to understand the tumor-immune cell interactions in the context of heterogeneous TME, focusing on the role of tumor cell HLA-1 expression as a modulator of immune cell infiltration, using samples from metastatic melanoma patients. The Cell DIVE technology enables phenotypic single-cell characterization of many markers while preserving the spatial micro-anatomic location of the cells within the sample. As such, the combined data allows interrogation of spatial relationship based on cellular phenotypes using our analytical algorithms. This approach enables quantitative and spatial modeling of cellular distribution and interplays between tumor and immune cells. Using these tools, we were able to demonstrate that HLA-1 tumor cell expression is highly heterogeneous both within and across tumor cells of similar morphology. The level of tumor HLA-1 expression heterogeneously correlates with the magnitude, nature and pattern of infiltrating immune cells (TIL and others), with unique cellular distributions and interaction patterns between areas with brisk vs. non-brisk T-cell infiltration. Importantly, this study establishes a novel approach to analyze the large amount of data obtained from multiplexed imaging system, and to model the cellular interactions and distributions at both single cell and TME level, allowing the elucidation of the “homogeneous patterns” within the heterogeneous TME that can impact the clinical outcome of tumor immunotherapy.

## Materials and methods

### Clinical cohorts

The primary objective of this study was to develop mathematic and spatial analytic tools to investigate the relationship of melanoma tumor cells, focusing on their HLA class I (HLA-1) expression levels, and the composition of the surrounding immune cell response within the TME. The effect of melanoma HLA-1 expression on clinical and treatment outcomes was also investigated. Four excisional metastatic lymph node biopsies were selected for Cell DIVE methods, obtained from metastatic melanoma (MM) patients who received no systemic therapies. Key Cell DIVE findings were confirmed by tumor microarray (TMA) using traditional immunohistochemistry methods. For TMA, we selected 173 tumor cores of lymph node metastases obtained from 158 patients with stage III melanoma, and all patient clinical information was de-identified. Among these patients, a total of 138 patients with complete clinical follow up were included in the survival analysis. This study was approved by the Mayo Clinic Institutional Review Board of Rochester, Minnesota. Informed consent was obtained from all patients.

### TMA production, immunohistochemistry, and scoring

A TMA was created from 173 archival paraffin blocks of MM using a Galileo Tissue Microarrayer (Integrated Systems Engineering SRL). Duplicate 1-mm tissue cores were removed from the donor blocks and placed in 2 recipient blocks. Tissue cores were randomly selected from the blocks to account for tissue heterogeneity. For spatial orientation and controls, additional cores of liver, placenta, and paraffin were placed in the recipient blocks. Immunohistochemistry was performed on tissue sections from the TMA block with the antibodies listed above, using heat-induced epitope retrieval and a platform detection system (BenchMark XT; Ventana Medical Systems, Inc). TMA slides were scored qualitatively on a scale of 0 to 3+ for HLA-1 and CD8. Samples with both analyzable HLA-1 and CD8 staining were included in further analysis. Fourteen patients in this study had >1 tumor samples (13 with 2 cores and 1 with 3 cores) selected for developing the TMA.

### Antibodies and reagents

Antibodies used in Cell DIVE multiplexed immunofluorescence (MxIF) are summarized in S1 Table. Commercial antibodies were purified and conjugated with either Cy3, Cy5, or Cy7 as previously described [14, 16].

For the TMA analysis antibodies included anti-HLA-1 (Abcam catalog No. ab52922), anti-CD8 antibody (Ventana Medical Systems, Inc, catalog No. 790–4460, Clone SP57), anti-PD1 antibody (Abcam catalog No. ab52587, Clone NAT105), and anti-CD20 antibody (Dako catalog No. M0755, Clone L26) in TMA analysis. These were stained using routine IHC DAB detection on sequential sections using a DAB on a Ventana autostainer (Ventana Medical Systems).

### In situ immune cell phenotyping and quantification with CellDIVE

Ten archival MM samples were applied to MxIF microscopy in accordance with previously reported protocols [14]. Briefly, sections were reviewed by a pathologist through hematoxylin-eosin staining before inclusion for analyses. Samples were cleared of paraffin, 4,6-diamidino-2-phenylindole (DAPI) stained, and scanned using a 10X objective using filter cubes each for DAPI while Fluorescein isothiocyanate (FITC), Cyanine-3 (CY3), and Cyanine-5 CY5 channels were collected for autofluorescence signal. This first scan was stitched and converted into a psedo-colored haematoxylin and eosin stain (virtual H&E) image similar to hematoxylin-eosin. From the whole tissue scan approximately 20 fields of view (FOV) were selected from each sample capturing both central and peripheral regions of the tumors. Tissue slides then underwent iterative cycles of staining and 20X imaging until all markers of interest were addressed (21 markers in this study). The images were processed through illumination correction, registration and autofluorescence removal prior to performing single cell segmentation and quantification as described [14]. For this study, the cytokeratin antibodies typically used were substituted with anti-S100 to mark the tumor cell population and to create the tumor segmentation mask. A final review of the captured images was done to ensure the fidelity of the samples through the iterative process, and that the segmentation algorithm had performed correctly.

An algorithm for immune cell identification and quantification was applied that used multimarker information to build Support Vector Machine (SVM) models to classify different cell subtypes [17–19]. A training data set was generated to build SVM models based on the specific expression patterns. After the cells were classified, the algorithm generated coded prediction overlays that showed not only the predicted cell types but also the probabilities for that assignment. These images were used to visually verify algorithm performance. In addition, user annotations where specific cell populations were marked in the images were used to generate sensitivities and specificities that, for most cell types, were >90%.

The output files produced by MxIF technology contain X and Y coordinates in pixels of the cell centroids, fluorescence intensity for each immunostain in individual cells and subcellular compartments, cell morphological features (e.g. cell ellipticity, the nuclear, membrane, cytoplasmic, and cell area, and the number of nuclei per cell) and multiple metrics for quality control (QC). QC metrics per cell include the level (range 0–1, 0 being no alignment and 1 meaning perfect alignment) of alignment of cell image in each round of immunostaining with the original (baseline) DAPI image of the nucleus as well as the level of alignment through all rounds. Before data analysis, virtual hematoxylin-eosin and segmentation images were visually assessed for tissue and segmentation quality, and only high-quality images were included in the analysis. Within the selected images, cells were filtered to exclude poorly aligned, segmented, or artificially generated cells using QC criteria. A QC value of 0.85 was used for nuclear alignment (available range, 0–1; 1 for full alignment and 0 for no alignment), each cell must contain ≥10 pixels per compartment (membrane, cytoplasm, and nucleus) and ≤2 nuclei per cell. Cell coordinates were converted from pixels to microns.

An advanced version of the Cell-DIVE platform with an order of magnitude faster imaging, automated image processing and improved machine learning based immune cell analysis workflow is now commercially available through GE Healthcare, catalog number 29266690.

### HLA-1 and immune cell analysis in MxIF

To determine HLA-1 status of the melanoma, cells in the S100 tumor mask from all 4 excisional biopsies were analyzed for HLA-1 expression. To classify a tumor cell as HLA-1 positive, we used fluorescence value cutoff based on an HLA-1 signal greater than the 10% of the HLA-1 fluorescence intensity of CD3+ cells across all regions of interest (ROIs) (The HLA-1 expression levels on CD3+ were used as internal controls since HLA-1 is ubiquitously expressed on CD3+ cells). Immune cell subtypes were classified using SVM models built on the basis of expected staining patterns for each phenotype. For example, to classify T and B cells, models were built for the expected mutual exclusivity of CD3 and CD20 marker expression in these cells. Similarly, coexpression of CD4 and FOXP3 was used to define CD4 Treg.

### Spatial analysis

Analysis of spatial patterns was performed using SpatStat package. For analysis of cell-cell attraction or repulsion, we developed an algorithm named cell neighborhood analysis (CNA). This algorithm uses SpatStat function to traverse through every point on a 2-dimensional plane and create a neighborhood of a requested size R. CNA counts all types of points (ie, cells) in each neighborhood and records coordinates and counts in a matrix. In this study we counted lymphocytes in the vicinity of tumor cells for neighborhood sizes from 2 to 50 μm. The analysis showed the optimal neighborhood size to be 12 μm. Other R tools (ie, ggplot2, maptools, rgeos, plyr, and pastecs) were also used as needed for statistical/spatial analyses.

### Statistical analysis

All spatial analyses were performed in R programming language. To assess the difference of cellular marker expressions (e.g., HLA-1, CD3) among patients, all cells with that specific marker from all biopsy ROIs were included, and Welch t test was applied to compare median expression levels. For visual representation, the percentage of each cell population within the ROI was first calculated; a color scale then was applied to convert cell counts to color analogous to the technique for heat map construction. For survival analysis, we used statistical software (JMP version 10.0.0; SAS Institute Inc). Progression free survival (PFS) and overall survival (OS) were evaluated using the Kaplan-Meier method. Statistical significance was defined as P value < .05.

## Results

### 1. Tumor heterogeneity in melanoma TME

The presence of different types of cells with various distributions is one of the important features of TME heterogeneity. To examine the heterogeneity of tumor cells in metastatic melanoma (MM) tissues, we examined the tumor HLA-1 expression levels between MM samples obtained from different patients. Previous in vitro experiments performed in melanoma cell lines have shown ubiquitously high HLA-1 mRNA expression [20]. Here, we investigated tumor HLA-1 expression in 166 resected metastatic lymph node samples obtained from patients with stage III melanoma. A composite tumor microarray (TMA) was immunostained for HLA-1 and scored by our pathologist using an established international scoring system [21]. High expression of HLA-1 (graded as 3+) based on pathologic review was observed in about two-thirds of the cases (S1 Fig). Although TMA only randomly reflects part of the entire heterogeneous TME, this result directly confirms the interpatient heterogeneity of melanoma HLA-1 expression in a larger sample size.

To further explore the tumor heterogeneity at the TME level, we then investigated the intratumoral heterogeneity of tumor cell HLA-1 expression using Cell DIVE on FFPE excisional biopsies obtained from untreated MM patients, 15–20 fields of view (FOVs) per biopsy, to represent tumor and peripheral regions of TME). Our data showed that different regions within the same TME contained tumor cells with different levels of HLA-1 expression (S2A Fig). This finding was observed consistently in all biopsies, suggestive that MM HLA-1 expression is highly variable within the tumor. Next, we examined tumor cell HLA-1 expression in biopsies from different patients. Again, MM tumor cell HLA-1 expression was highly variable among patients (S2B Fig).

Decreased expression of tumor HLA-1 presumably associates with impaired tumor antigen presentation and decreased cytotoxic T lymphocytes (CTL) activation [10, 22–24]. We next examined the CTL infiltration into the heterogeneous tumor using the same TMA set (S1 Fig). Immunohistochemistry involved staining for CD8 and was scored by an experienced pathologist. High score of CD8+ TILs (graded as 3+) is almost always seen only in tumors with high HLA-1 expression, although some HLA-1 high-expressing tumors contain low levels of CD8+ TILs (S1B and S1C Fig), suggesting that tumor HLA-1 expression is required but not sufficient for CD8+ cell infiltration in TME.

Next, we examined the effect of heterogeneous tumor cell HLA-1 expression on infiltrating immune cells at the TME level using Cell DIVE. When comparing high HLA-1 tumor-containing TME with low HLA-1 tumor-containing TME, we observed differences in CD4+ and CD8+ TILs (S2C Fig). Interestingly, PD1 analyses showed that most CD8+ TILs invading HLA-1 high tumor areas were also PD1+, suggesting susceptibility to PD-L1 inactivation. These results showed that functional profiles of tumor cell and T cell tumor infiltration within the TME are highly heterogeneous.

TME also contains various other types of immune cells, playing different roles in anti-tumor immune response. Taking advantage of multiplexed image system, the expression levels of different immune cell markers can be easily examined simultaneously, not only in the tumor-containing area, but also the immune cell–rich regions surrounding the tumor area. As shown in S2C Fig, the composition and distribution of different immune cell types are extremely heterogeneous between different micro anatomic locations from the same patient and between patients. A considerable amount of CD20+ cell accumulation was seen surrounding/approximating the regions with HLA-1 low-expressing tumor cells, with few infiltrating T cells. Additionally, some of the CD20+ cells also expressed PD1, indicating its potential immunosuppressive role (S2D Fig) [25]. Also, HLA-1 low-expressing tumor areas appeared to frequently be surrounded by a greater number of CD4+FOXP3+ (regulatory T cells) and a lower number of CD68+ (likely macrophages) immune cells relative to HLA-1 high-expressing tumor regions of the same TME (S2C Fig). Taken together, these results clearly demonstrated the intra- and inter-tumoral heterogeneity of tumor HLA-1 expression levels within the metastatic melanoma TME, which can potentially impact the various immune cell distributions.

### 2. Quantitative assessment of melanoma TME cellular heterogeneity

In addition to images for direct visualization, Cell DIVE provides large amount of single-cell based marker-expression data for quantitative assessment, and mathematical algorithms can be subsequently applied for statistical analysis, which provides direct measurements elucidating the cellular features in TME.

**Fig 1:**
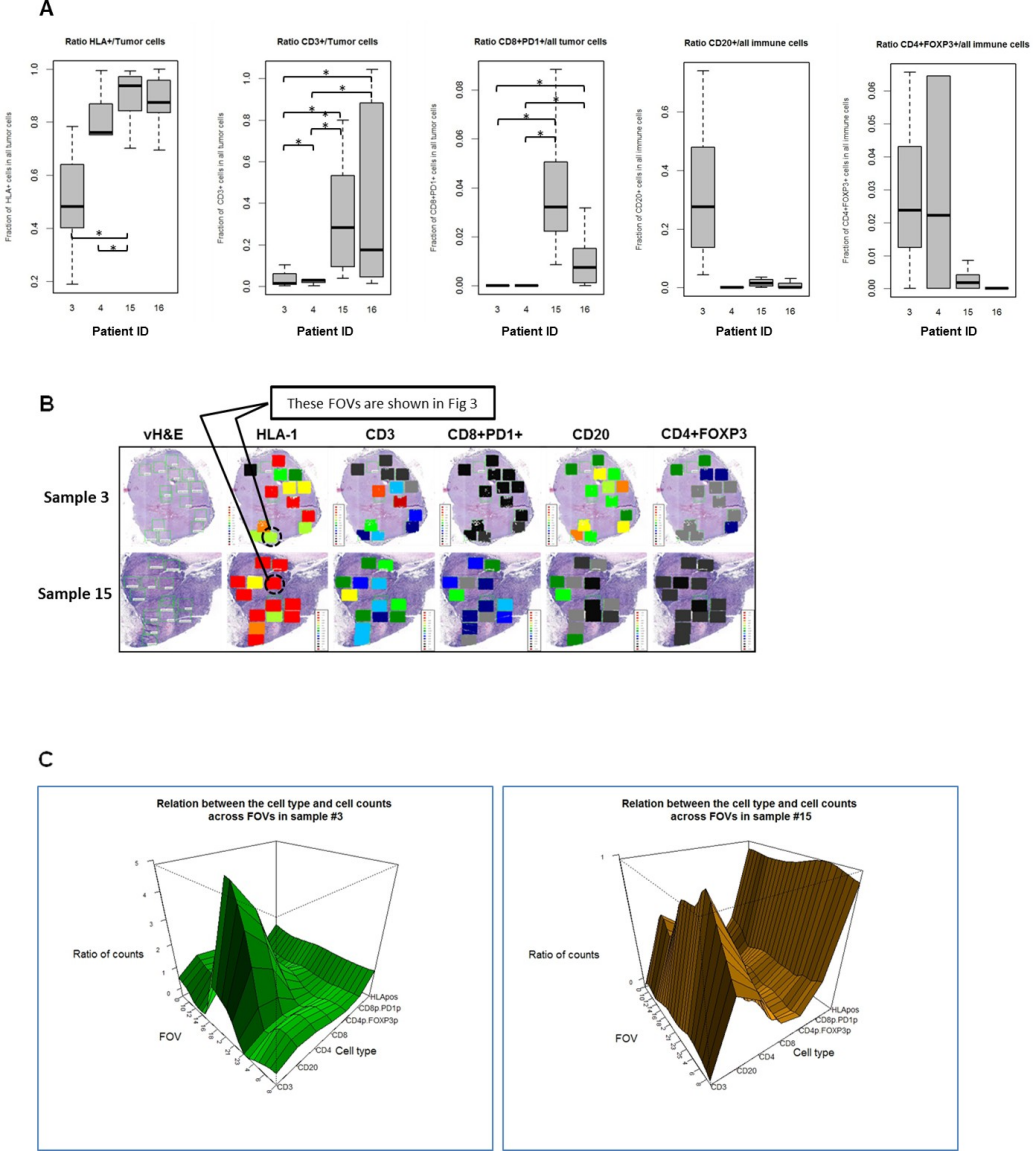
Quantitative assessment of melanoma TME cellular heterogeneity. A. Ratios (y-axis) of counts of different cell sub-types to the counts of tumor cells or all immune cells per tumor sample. Box plots represent the range (lower edge– 25 percentile, middle– 50th percentile and top edge– 75th percentile) of ratios across all FOVs for each tumor sample. Shown here are ratios for HLA1+ tumor cells/all tumor cells, tumor infiltrating CD3+ cells/all tumor cells, tumor infiltrating CD8+PD1+ cells/all tumor cells, CD20+ cells /all immune cells, CD4+FOXP3+ cells/all immune cells. X-axis indicates different sample IDs. Horizontal bars with asterisks represent comparison where the difference was significant with p-value<0.05. B. Heterogeneous Melanoma HLA-1 Expression Associates With Distinct Heterogeneous Distribution of Different Immune Cell Subsets in Tumor Microenvironment. Multiple regions of interest from metastatic lymph node excisional biopsies of 2 patients were selected (green boxes shown in vH&E images) and applied to MxIF for multiple cell markers. Heat maps represent the ratios of HLA-1–expressing melanoma cells to total melanoma cells; tumor-infiltrating CD3+ cells to all tumor cells; tumor-infiltrating CD8+PD1+ cells to all tumor cells; tumor-surrounding CD20+ cells to all tumor-surrounding immune cells; and CD4+FOXP3+ cells to all tumor-surrounding immune cells. HLA-1 indicates HLA antigen 1; PD1, programmed death protein 1; vH&E, virtual hematoxylin-eosin. C. 3D plots for the ratios of cell counts to the number of tumor cells (HLA-1, CD3, CD4, CD8, CD8+PD1+) or immune cells (CD20, CD4+FOXP3+). 3D plots show ratio of counts (Z- axis) of 7 types of cells (Y-axis) to the number of tumor cells or total number of immune cells for each FOV (X-axis) in sample 3 (left panel) and 15 (right panel) (calculated as in Fig 1B).

One of the current approaches for TME analysis is quantitative cellular measurement. With the cell-level marker data with associated anatomic location, we were able to quantitatively assess the tumor expression of HLA-1 by counting HLA-1 expressing tumor cells in 80 FOVs from 4 excisional biopsies processed with CellDive. The fraction of HLA-1 expressing tumor cells are highly variable across all FOVs within each TME and are significantly different when median values obtained from all FOVs within a sample were compared between 4 samples (Fig 1A). This approach allows us to mathematically quantify the inter/intra-TME HLA-1 heterogeneity and to classify TME and FOVs as high HLA-1 or low HLA-1.

We also quantified the number of cells expressing each of the following makers, CD3, CD20, CD8, PD1, CD4 and FOXP3, in the same FOVs. This analysis showed that fraction of each cell type presented in the TME varies between FOVs and samples (Fig 1A). We observed differences in CD3+ and CD8+PD1+ TILs between HLA-1 high vs. low tumors were statistically significant (Fig 1A). HLA-1 high tumors showed higher TIL infiltration, while HLA-1 low tumors contain high CD20+, and high CD4+FOXP3+ populations in the TME.

Interestingly, the variabilities in tumor HLA-1 expression levels and immune cells distributions were seen in 2 different tissue samples obtained from the same patient (sample 4: primary melanoma lesion; sample 3: melanoma lymph node metastasis), demonstrating the consistent TME heterogeneity in different anatomic sites and at different stage of disease progression, which needs to be further evaluated in future larger cohort of prospective clinical studies.

For samples 3 and 15 (both lymph node metastases), the quantified levels of each cell type were then represented at the tumor level in the forms of heat map and 3D map for direct visual investigation and assessment (Fig 1B and 1C). Although there are general associations between tumor HLA-1 expression level and both the pattern and magnitude of immune cells distributions (e.g. high HLA-1 tumor contains higher TIL, and low HLA-1 tumor contains higher CD4+FOXP3+), these associations are clearly heterogeneous even at the TME level. For example, FOVs with similar tumor HLA-1 expression levels clearly differ in their levels of TILs and CD20+ cells (Fig 1B and 1C), consistent with our TMA findings that high HLA-1 tumors contains variable levels of TILs. These findings suggest that the underlying dynamic cellular interactions and regulatory network between tumor and immune cells drive such phenotypic differences, therefore, variable outcomes of antitumor immune responses.

**Fig 2:**
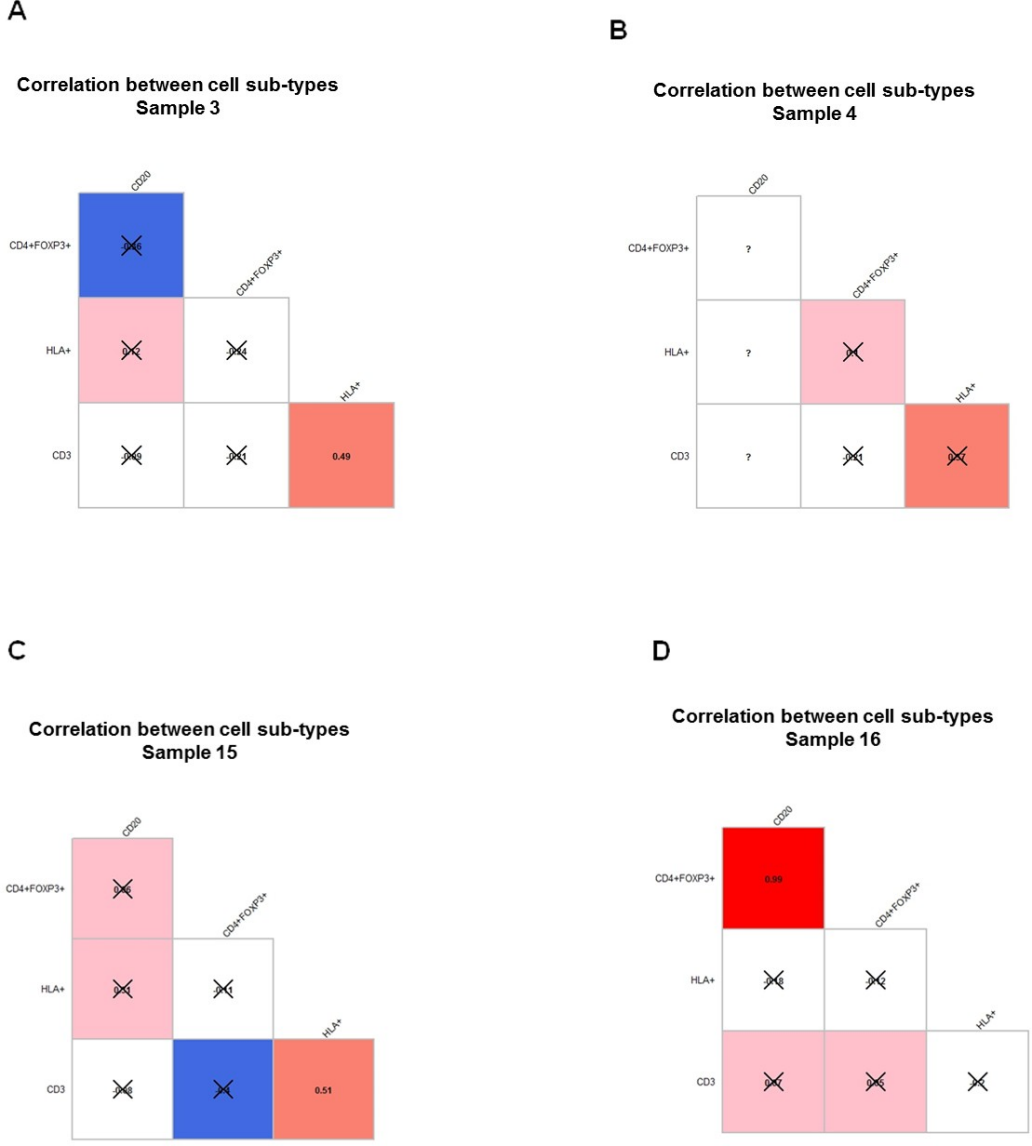
Correlograms demonstrating correlations between ratios of cell counts shown within each FOV from all four patients samples. A-D. Same samples as in Fig 1 were used. Pearson r coefficient between ratios of cell counts was computed across all FOVs within each tumor sample in each sample. The value of correlation coefficient is shown in each box and represented by the color in increasing order: blue, white, pink, red. Blue represents negative correlation coefficient and inverse correlation. P-value was calculated for each correlation coefficient and crossed boxes show correlation which did not pass the 0.05 cutoff of p-value. Question marks correspond to cases with insufficient data. Correlations that are not statistically significant are X-ed out.

To determine whether the presence and levels of different immune subpopulations in the TME are directly associated with each other, the correlation coefficients between the ratios of each cell type were examined across FOVs from each sample. As shown in Fig 2, no consistent significant correlations were observed across all FOVs from each sample. The only exception is that the ratio of HLA-1 expressing tumor cells was correlated moderately (r~ 0.5) with the ratio of CD3 T cells in the TME in sample 3, 4 and 15, while this correlation was not significant in sample 4. These findings demonstrated the limitation of quantitative assessment using overall cell counts in the study of cellular interaction in melanoma TME, and underscore the needs of developing in-depth analytical tools at single-cell level to identify micro-anatomic regions with specific biologic relevance and to reveal the pattern and regulation of spatial distributions at single cell level.

Given the immune cell heterogeneity in the TME, this result also suggested that the interaction between CD8+ T cells and tumor cells could be impacted by their micro-anatomic locations and the presence of other types of immune cell in the TME in a dynamic fashion. Developing spatial analytical tools and applying them in TME with different features, such as brisk vs. non-brisk CTL infiltration areas, will be crucial to understand the regulation of these interplays in the TME.

Taken together, our findings suggest a potential underlying link between the magnitude of tumor cell HLA-1 expression and the nature of tumor-associated immune response in TME. Although the quantitative calculation of marker expression levels can be easily achieved, only when it is combined with spatial information one will be able to further dissect the immune cell distribution patterns and their biological impacts.

### 3. Algorithm for quantitative analysis of cell aggregates

The heterogeneity of cellular composition and distribution in the TME suggests that the anti-tumor immunities in different micro-anatomic locations within the same TME are various, which could lead to different responses to therapy and ultimately clinical outcomes. For example, tumor areas heavily infiltrated by CD8+PD1+ T-cell are likely to response to PD-1 blockade, relative to other TME regions. Therefore, recognizing specific anatomic locations with specific features within TME and analyzing the underlying cellular spatial and functional interactions in these areas may provide critical biologic insights to identify the key features impacting the outcomes of anti-tumor immune responses.

**Fig 3:**
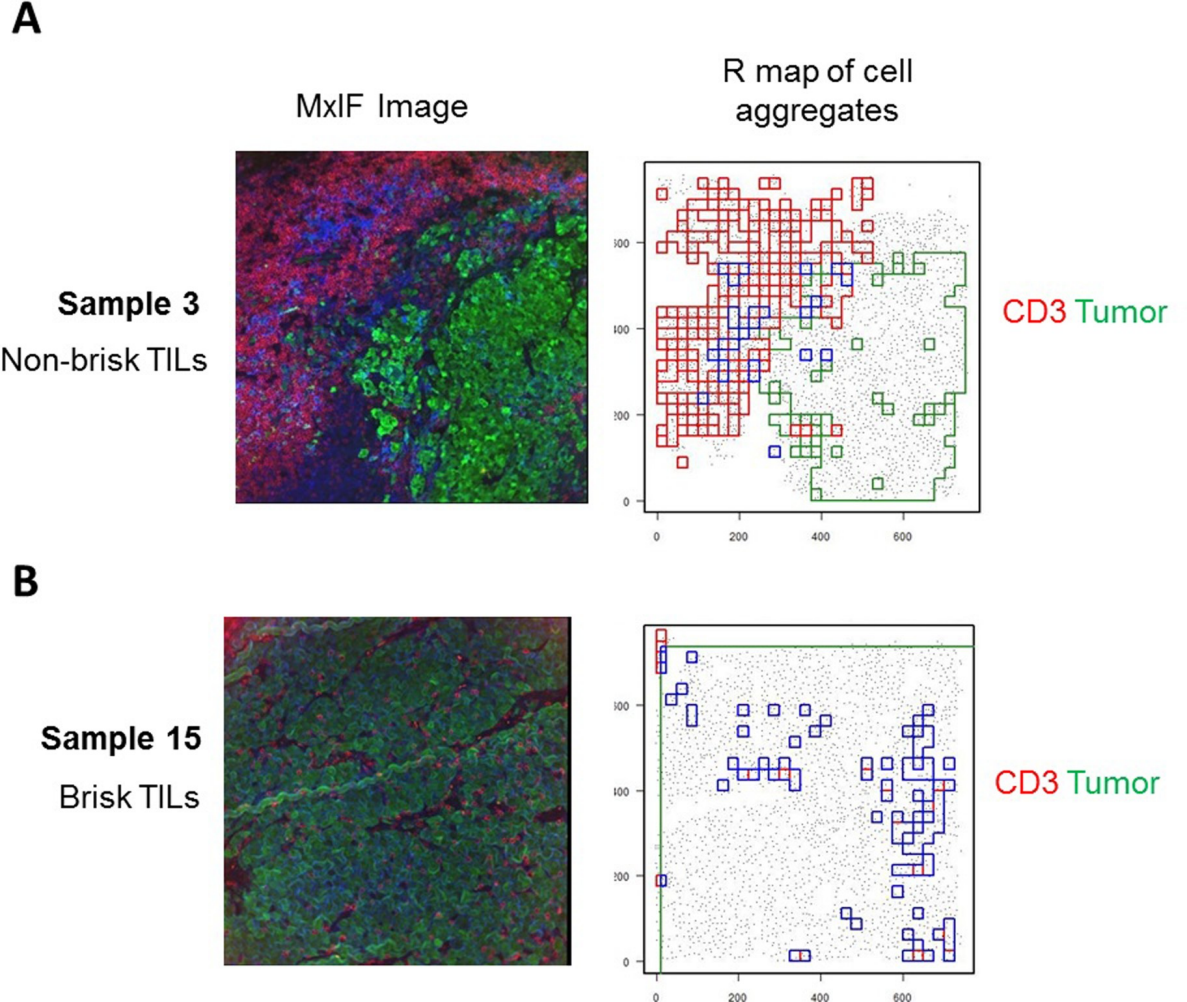
Cell aggregation algorithm (CAA) delineates areas of cell aggregates as polygons of complex shape. The MxIF images of two exemplar FOVs (seen in Fig 1B) are shown here. A corresponds to the tumor with non-brisk T cell infiltration, while B corresponds to the tumor with brisk tumor T cell infiltration. MxIF images shown on the left (tumor cells (green), CD3+ cells (red)). R maps (shown on the right) demonstrate aggregates of tumor cells (green), CD3+ cells (red) and intersection of these regions (blue).

We used methodology and algorithms developed in the research of spatial patterns in ecology [26–29] to analyze spatial relationships between different cell types in different anatomic regions (such as inside and outside T-cell infiltrated tumor area) within a TME. Based on the established methods in the SpatStat package for R programming language [29], we developed a custom **cell aggregation algorithm (CAA)** for the analysis of cell aggregates in a 2D space. This algorithm works in the following 7 key steps: i) subdivide the whole study area into squares of equal size (grid); ii) count cells of each type in each square; iii) compute distribution of cell counts across all squares; iv) locate squares with counts above specified percentile (e.g. 95) of the distribution from previous step. These squares become centers of a cell aggregate; v) walk around such a square, one layer of squares at a time, and add squares with a pre-defined number (cutoff) of cells in them to the expanding cell cluster; vi) stop when squares adjacent to the growing aggregate (cluster) contain fewer cells than pre-defined cutoff; vii) contain all joint as well as disjoint squares in a polygon. The resulting polygon may have either a very simple and compact or a very complex shape (Fig 3) depends on the marker selected for the analysis. This algorithm enables the generation of polygons that reflect the spatial distribution of cell type(s) with specific feature(s) within the TME, allowing further spatial analysis.

### 4. Quantitative analysis of cell aggregates using CAA

In order to select tumor areas with T-cell infiltration within the TME, we applied the CAA to identify aggregates of CD3+ cells and tumor cells. We then identified areas of intersection of these two different types of cell aggregates. These intersections represent areas of CD3+ T-cell infiltrating areas (IAs) in the tumor (Fig 3A). Since T-cell infiltration directly associates with better clinical outcome, identifying these regions in the TME is of special importance, allowing us to understand the unique feature in these areas and to evaluate the key regulators orchestrating the T cell infiltration.

**Fig 4:**
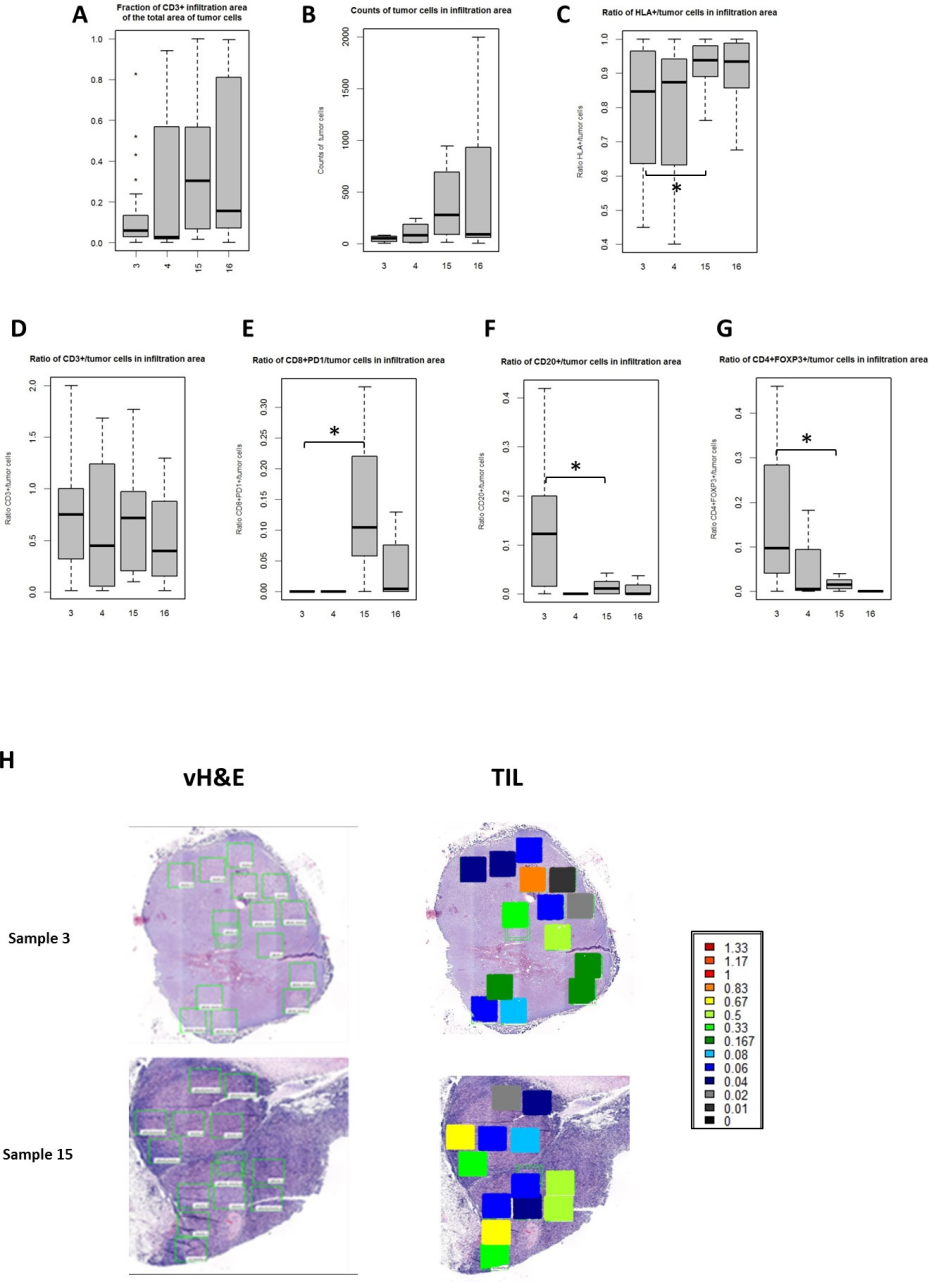
Boxplots showing the range of the ratios of counts of different cell sub-types to the counts of tumor cells or all immune cells per tumor sample in the Infiltration Areas (IAs). A-G. Box plots represent the range (lower edge– 25 percentile, middle– 50th percentile and top edge– 75th percentile) of ratios across all FOVs for each tumor sample. A. ratio of the area of CD3+ IA to the whole area of tumor cells; B. counts of tumor cells in IAs; C. ratio of HLA+/tumor cells in IAs; D. ratio of CD3+/tumor cells in IAs; E. ratio of CD8+PD1+/tumor cells in IAs; F. ratio of CD20+/tumor cells in IAs; G. ratio of CD4+FOXP3+/tumor cells in IAs. Horizontal line with asterisk shows statistically significant difference. H. Heat map of the two exemplar tumor samples showing the level of CD3+ T cell infiltration in the tumor in the IAs in each individual FOV from 2 patient samples. The color scales indicate the ratios of T cell counts per tumor cells.

Next, we assessed the size and cell composition of IAs by counting cells of 6 different sub-types (all tumor cells, HLA+ tumor cells, CD3+, CD4+ FOXP3+, CD8+PD1+, CD20+ cells) and calculated counts of each of these subpopulations relative to the number of total tumor cells in IAs (Fig 4). This analysis was performed for each of 80 FOVs from all 4 samples that we studied. The average size of IAs in sample 15 and 16 was 316321 um^2^ and the average fraction of tumor areas infiltrated by CD3+ cells was 0.38. In samples 3 and 4, the average size of IAs was 319930.2069 um^2^, while the average fraction of CD3 infiltrating-tumor area was 0.17, significantly different compared to that of samples 15 and 16 (p-value = 0.004, Cochran-Mantel-Haenszel test). We refer to samples 15 and 16 as tumors with brisk TILs and samples 3 and 4 as tumors with non-brisk TILs (Fig 4A).

We also calculated counts of each of these cell subtypes per each tumor cell (Fig 4B–4G) and applied Cochran-Mantel-Haenszel test to assess the significance of the difference between a tumor with non-brisk IAs and a tumor with brisk IAs. Heat map demonstrating the levels of IAs from two samples are shown in Fig 4H. The level of T-cell infiltration into tumor varies across FOVs within and between each tumor sample (represented as ratio of infiltrating area size to total tumor area size), with coefficients of variation is 0.45 for non-brisk IAs and 0.53 for brisk IAs.

**Fig 5:**
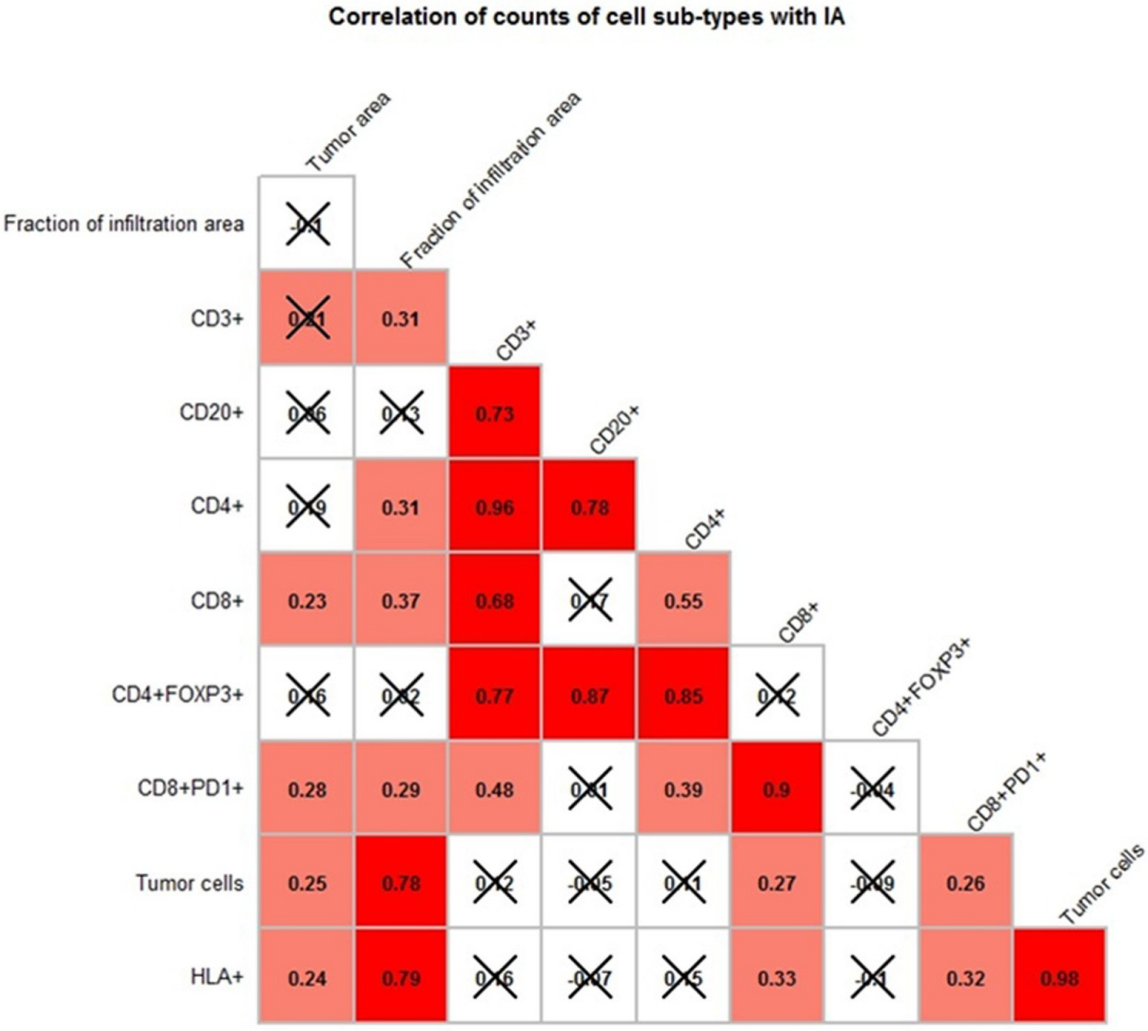
Correlogram demonstrating correlation between ratios of cell counts within each FOV within the Infiltration Areas (IAs). Pearson r coefficient between the number of cell counts in IAs were computed across all FOVs from 4 tumor samples. The value of correlation coefficient is shown in each box and represented by the color in increasing order: blue, white, pink, red. Question marks correspond to cases with insufficient data. Correlations that are not statistically significant are X-ed out.

To better understand the interplay between immune subsets within the IAs, the correlations between different cell types were examined across all IAs (Fig 5). The numbers of all tumor cells and HLA-1+ tumor cells correlated significantly with the fraction of the IA in the whole tumor area, demonstrating that tumor areas with more brisk T-cell infiltration is associated with tumor HLA-1 expression. The correlations between other cell populations are shown in Fig 5. As expected, the association between CD3+ and CD4+, CD3+ and CD8+ cells are also observed. In addition, correlations between CD20+ and CD4+FOXP3+ were seen, implying their potential functional associations within the TME that may negatively impact the infiltration of immune cells into tumor areas.

In conclusion, this quantitative cell aggregate-based analysis demonstrates the ability to visualize and to analyze the complex cellular interplays in specific TME regions with distinct biologic and immunologic features, enabling the selection of key cellular features that can impact the outcome of the anti-tumor immune response, such as T cell infiltration.

### 5. Algorithm for single cell analysis

We showed that by selecting micro-anatomic regions with distinct biologic features, unique cellular composition and association can be observed. However, to further dissect the spatial interaction and regulation network within these regions, single cell-based analytic tools need to be established.

**Fig 6:**
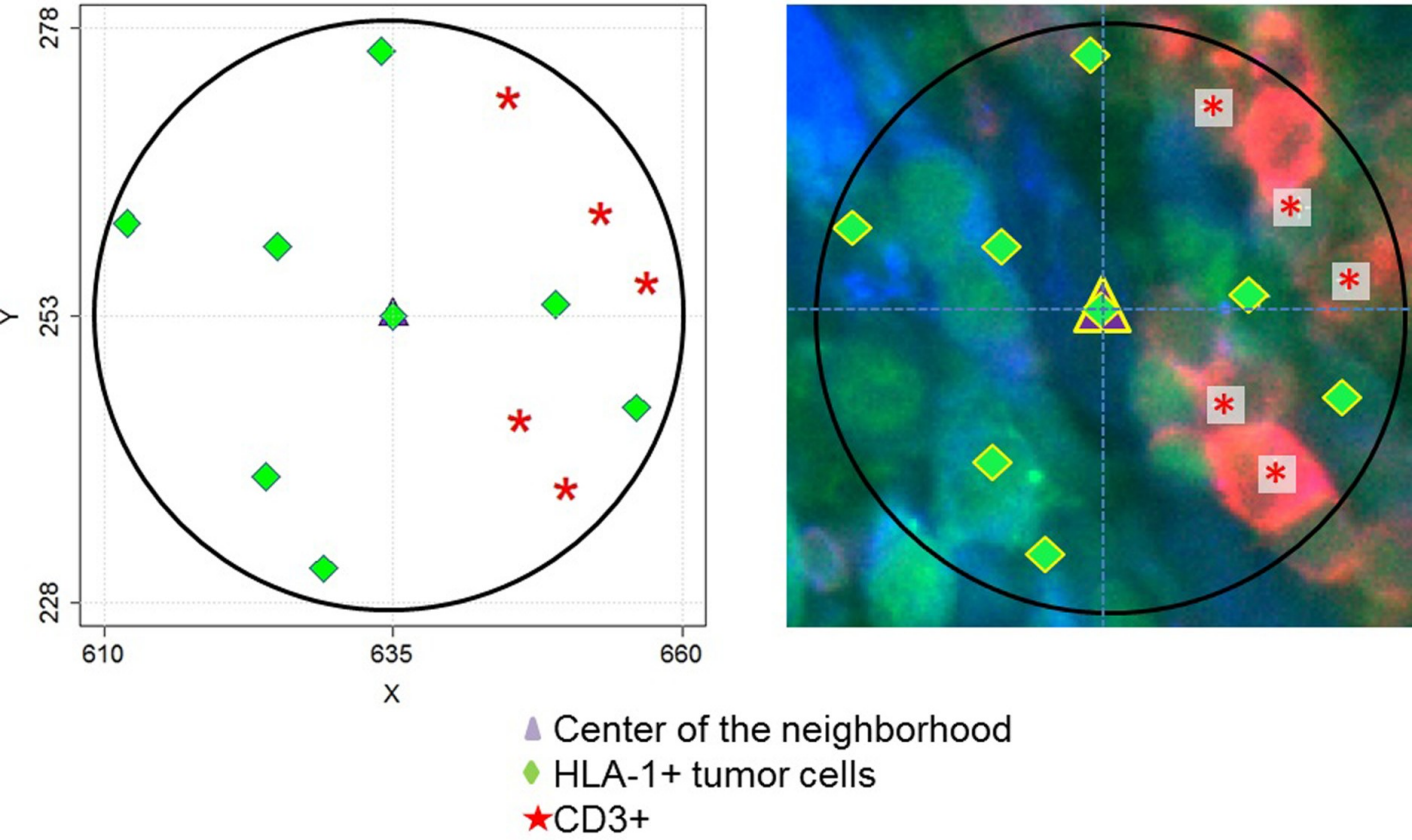
Cell neighborhood analysis algorithm (CNAA). The map on the left demonstrates an example of a single neighborhood of a cell (purple triangle). X and Y coordinates are shown in microns and the diameter of the neighborhood is 50 microns in diameter. Green diamonds represent positions of HLA1+ tumor cells and red asterisks represent positions of CD3+ cells in the neighborhood. The MxIF image of the same neighborhood is shown on the left. Red: CD3 marker; Green: HLA-1; Blue color: S100B.

Based on the established functions in the SpatStat package for R programming language, we developed a custom **cell neighborhood analysis algorithm (CNAA)** for the analysis of spatial relations between cells in microenvironments of individual cells. This algorithm sequentially visits every cell on a 2D plane and performs the following 4 key steps: i) records the markers present on the current cell; ii) creates a circular area (neighborhood) of a user- defined size surrounding the current cell; iii) counts cells of each sub-type in the neighborhood; iv) measures nearest neighbor distances (NND) between cells of user- defined types in the neighborhood (Fig 6).

### 6. Single cell analysis

**Fig 7:**
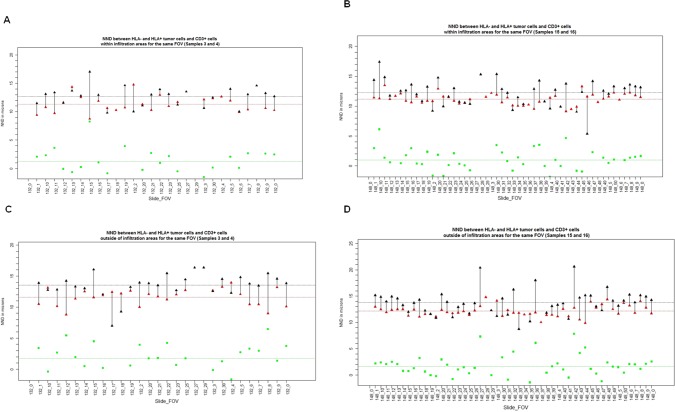
Distance between tumor and T cells. Distances between CD3+ cells and HLA+ tumor cells (red triangles) and HLA- tumor cells (black triangles) in the neighborhoods of CD3+ cells within IAs (A and B) and outside of IAs (C and D). A and C represents tumor samples with non-brisk TILs, and B and D represent tumor samples with brisk TILS. Each pair of red and black triangles connected by a line represent average distances for a single FOV shown on X–axis. Green squares represent the value of the difference between the two distances. Dotted lines represent average values of corresponding values (red, black and green) across all FOVs.

Next, we used **CNAA,** in combination with **CAA**, to assess the attraction and repulsion relations between various cells sub-types within IAs. We defined CD3+ T cells as the center of the neighborhood, and applied the CNAA within the IAs in all 4 samples. As shown in Fig 7, the nearest neighbor distance (NND) between CD3+ cells and HLA-1 + tumor cells is shorter than the distance between CD3+ cells and HLA-1- tumor cells in IAs regardless of the level of tumor T cells infiltration. In addition, HLA-1+ tumor cells are closer to CD3 cells within the IAs as compared to areas outside of IAs (11.17 um vs 12.16 um), suggesting that the attraction between HLA1 + tumor cells and T cells within IAs are unique to and consistent within infiltrated areas (Fig 7).

**Fig 8:**
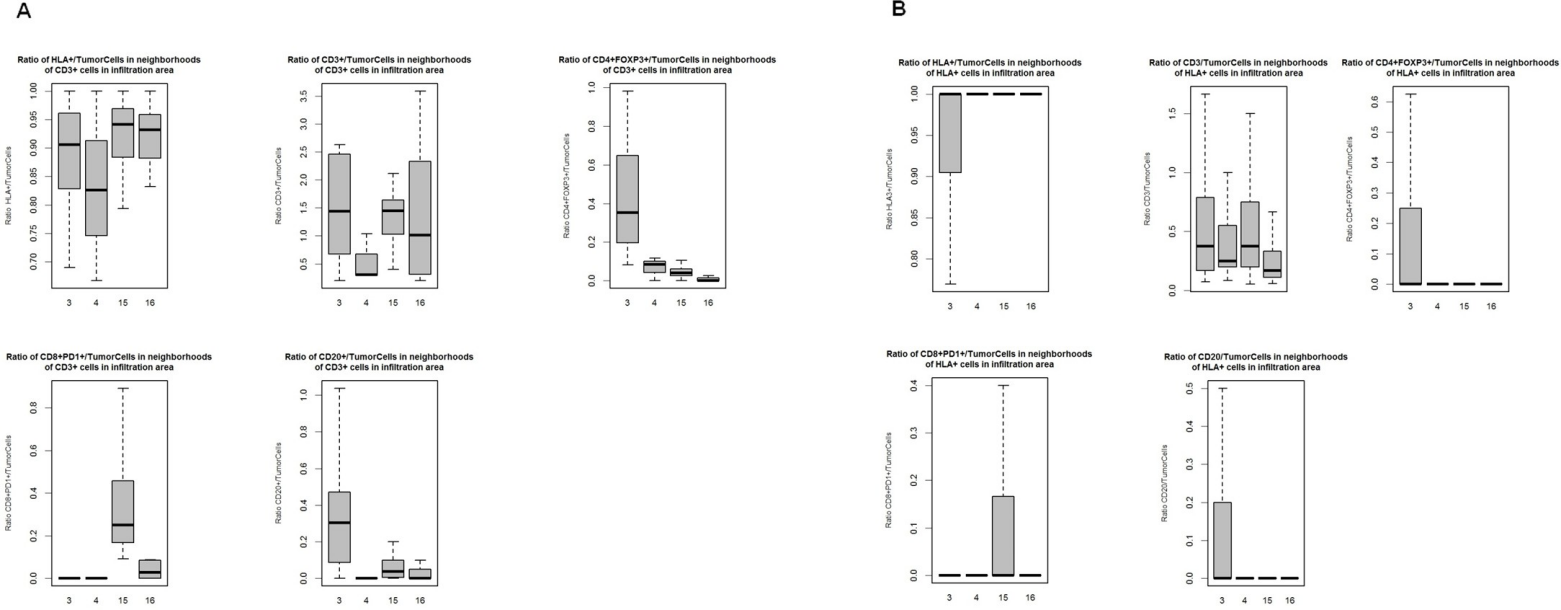
Ratios of different cell subtypes in the infiltration areas (IAs). Boxplots showing the range of the ratios of counts of different cell sub-types to the counts of tumor cells across all FOVs in the neighborhoods of CD3+ cells in the IAs (A) or in the neighborhoods of HLA+ tumor cells in the IAs (B). Each box plot represents the range of ratios across all FOVs for each tumor sample. A. Ratio of HLA+ tumor cells/all tumor cells, ratio of CD3+/tumor cells, ratio of CD4+FOXP3+/tumor cells, ratio of CD8+PD1+/tumor cells, ratio of CD20+/tumor cells in the neighborhoods centered by CD3+ T cells in IAs were shown. B. Ratios of HLA+ tumor cells/tumor cells, ratio of CD3+/tumor cells, ratio of CD4+FOXP3+/tumor cells, ratio of CD8+PD1+/tumor cells, ratio of CD20+/tumor cells in the neighborhoods of centered by HLA+ cells in the IAs were shown.

We also assessed the number of HLA-1+ tumor cells, CD3+, CD20+, CD8+PD1 and CD4+FOXP3+ cells, per each tumor cell within the CD3+ cell neighborhoods within IAs (Fig 8A). We found that more CD8+PD1+ T cells in IAs from tumors with more brisk TILs than those in IAs from tumors with non-brisk TILs, indicating more concentrated HLA-1 + tumor and T cells interaction within the immune-infiltrated tumor areas. In contrast, in IAs from less immune-infiltrated tumors, neighborhoods centered by CD3+ T cells contain higher numbers of CD20+ and CD4+FOXP3+ cells per each tumor cell and less HLA1+ tumor cells, favoring potential immunosuppressive environment. Similar trend holds true for neighborhoods centered at HLA1+ tumor cells (Fig 8B)

Our results showed that the combination of these two algorithms enables the single-cell level spatial analysis of cellular interactions between tumor and immune cells within sub-anatomic areas of TME. Unlikely quantitative methods, our approach allows the identification of potential underlying patterns of cellular interactions and the dynamic distributions of tumor and immune subsets, within a heterogeneous TME, that can affect the outcomes of anti-tumor immune response.

### 7. The impact of melanoma tumor heterogeneity on clinical outcomes

Studies have shown that HLA-1 expression level on melanoma cells can impact T cell tumor infiltration, therefore disease prognosis [30–32]; and a higher level of CTL tumor infiltration is known to be associated with better survival in multiple malignancies [33, 34]. Our data here demonstrated a potential underlying link between the level of tumor HLA-1 expression and CTL tumor infiltration. However, clear heterogeneities were observed not only in the expression level of tumor HLA-1, but also in its association with the magnitude of T cell infiltration, which can likely impact the prediction of clinical outcomes using HLA-1 alone. Moreover, the presence of other immune subsets with various immunemodulatory effects is also heterogeneous, which can further impact the success of anti-tumor immunity, and making single-marker based outcome prediction even more inaccurate, especially in fine needle aspirate (FNA)-based tissue analyses that only samples a small area of entire heterogeneous TMA. To examine this, we next explored the correlation between the level of tumor HLA-1, T cell infiltration and clinical outcomes. Complete clinical data were obtained from 138 patients with stage III melanoma with complete follow up data, whose samples were used for TMA experiment (S2 Fig).

Progression-free survival (PFS), defined as the time of stage III diagnosis to the time of progression, and overall survival (OS), defined as the time of stage III diagnosis to the time of death or last follow-up, were compared among patients with different levels of tumor HLA-1 expression and CD8 infiltration. We detected improved OS in patients with a high TIL level (score of 3) (median [95% CI] OS, 107 months [48 months to not reached]) compared with a low TIL level (median [95% CI] OS, 44 [28–65] months; P = .02) (S3A Fig). Interestingly, patients with high tumor HLA-1 expression (score of 3) also had significantly improved OS (median [95% CI] OS, 85 [41–115] months vs 29 [20–61] months; P = .03) (S3B Fig). Importantly, among patients with high HLA-1 tumor expression, the improved OS was only seen in the small set of patients who have high TILs (S3C Fig), suggesting that factors in addition to tumor HLA-1 expression level can impact the infiltration of T cells, therefore the clinical outcomes. However, these patients had no benefit in PFS (S4C Fig).

Lack of PFS benefit suggests that subsequent systemic therapy after disease progression may influence the OS. Therefore, we assessed the OS in the subset of patients who received systemic immune checkpoint inhibitor treatment (ie, pembrolizumab, nivolumab, and ipilimumab) after progression to stage IV disease. Among patients who received immunotherapy, high level of HLA-1 expression was associated with improved OS (S5 Fig); however, this improvement was not statistically significant (P = .065) given the small sample size (n = 28). No association was found between HLA-1 expression alone and the response rate to immunotherapy (S2 Table), which is consistent with previous reports [10]. These results are in line with our findings that tumor HLA-1 expression is a prerequisite, but is not sufficient, for CTL infiltration to TME. The association between tumor HLA-1 expression level of TIL is not linear, the complex cellular interaction within the TME should be considered in order to further understand the mechanistic regulation and outcomes of anti-tumor immune response. Our single cells based analytic approach can potentially provide further insight into these interaction and regulatory network by integrating large amount of cell expression data with spatial information in the setting of immunotherapy.

## Discussion

TME contains different types of immune cells that vary in quantity and spatial distributions. In addition, tumor cells can have different biological features, including their expressions of driver mutations, immune checkpoint molecules and antigen present machinery, which impacts their interactions with immune cells or other immune regulators presented in the TME. The underlying spatial relations and functional interactions between cell types will ultimately impact the outcome of antitumor immunity. This heterogeneity ultimately leads to variability in the outcomes of antitumor immune response, immune invasion versus immune evasion. With the recent advances in tumor immunotherapy, deep dive into the TME in the context of heterogeneity, especially the dynamic interplay between cells, will provide crucial information not only to predict response of the treatment, but also to overcome treatment resistance and improve overall outcomes. However, analytic tools that integrate cell level expression data and spatial information are currently lacking, and studies based on peripheral blood or homogenized tissue samples fail to preserve tissue context.

In this study, using CELL DIVE technology, we are able to quantitatively demonstrate the extensive cellular and phenotypic TME heterogeneity in samples obtained from metastatic melanoma patients. Moreover, we applied algorithms to the combined quantitative and spatial data obtained from CELL DIVE, which allows automated selection of anatomic regions of interest, such as T-cell infiltrated tumor areas, and dissecting features of cell interaction in these pre-specified regions. The cellular composition and spatial distribution within heavily T-cell infiltrated tumor areas are drastically different compared to minimally infiltrated tumor areas, even within the same tumor biopsy. We showed that tumor HLA expression favors T-cell infiltration; however the expression of HLA on tumor cells is not sufficient to support T-cell infiltration. Other infiltrating immune cell subsets, such as CD20+ B cells and CD4+ FOXP3 regulatory T cells also play an important role in determining magnitude of T-cell infiltration into the tumors.

The advantage of using multiplexed imaging system is that large amount of data on multiple cellular features can be obtained at once, yet this is also one of the main challenges in using these platforms, since the lack of efficient and appropriate analytical tools that can meaningfully incorporate the enormous amount of data. Hyperplexed immunofluorescence technologies like Cell-DIVE allow measurement of expression of multiple markers/pathways simultaneously in individual cells. Standard statistical tools, including univariate and multivariate models, cell clustering, have been applied to these data to generate meaningful correlations to clinical parameters. These correlations, however, do not hold true for all patients probably as they fail to include regional heterogeneity and cellular interactions (Figs 1 and 4). We believe this heterogeneity and interactions between different tumor and stromal cell types have significant effects of course of disease and its response to therapy.

To address these challenges, we introduced two algorithms that enable the deeper assessment of the cellular interplay within the TME. It allows for selection of any specific TME regions with particular biologic features, in a semi-automated manner, for single-cell based spatial analysis to dissect and compare distinct interaction features between different TME regions. The variability in clinical outcomes among patients treated with modern immune therapeutics may indeed be a function of such TME observed interactions. This analytical approach may enable discovery of underlying in situ biologic interactions that may explain clinical differences.

**Fig 9:**
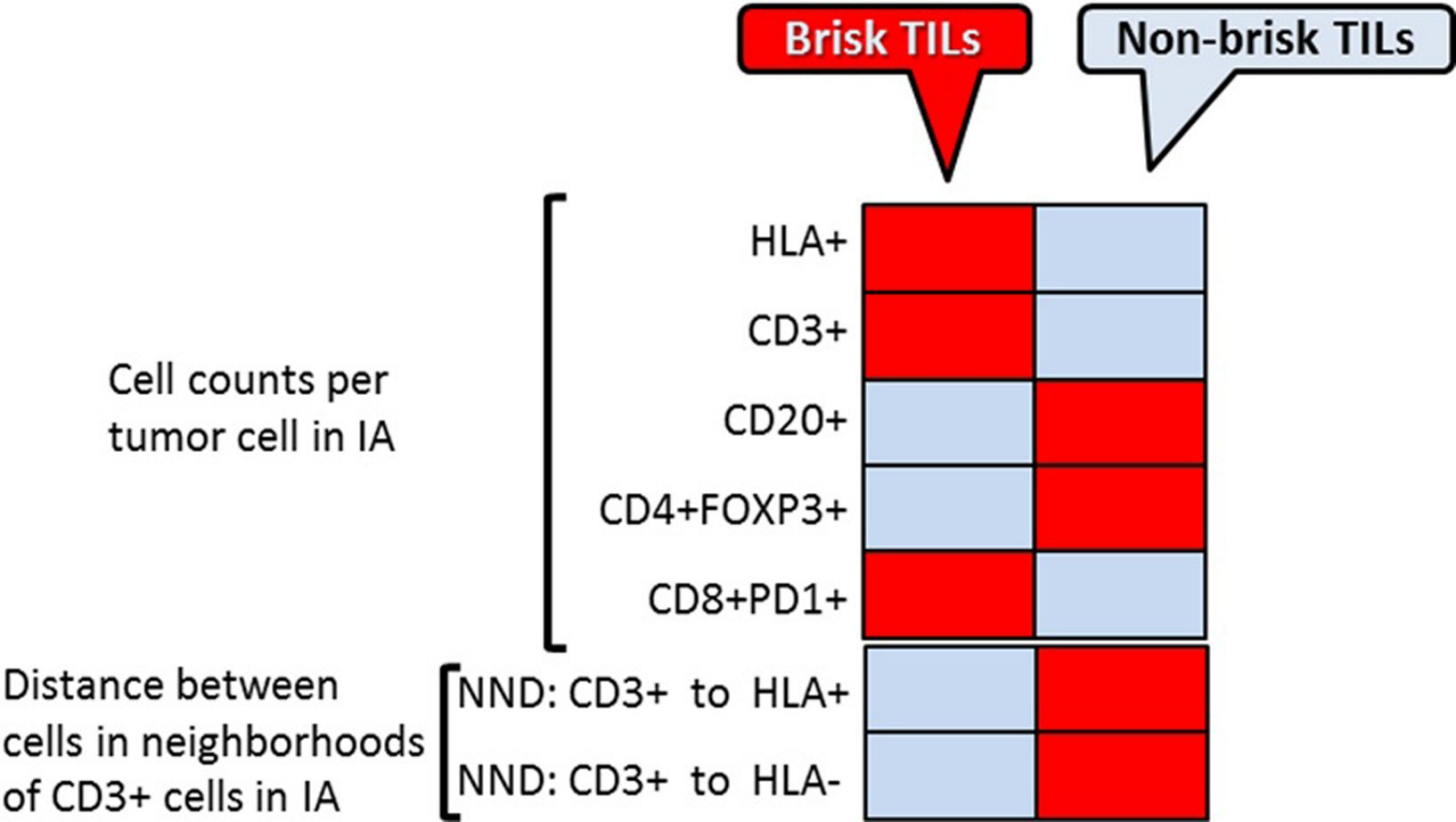
Spatial analysis of tumor-immune cells in melanoma TME. Heat map summarizes the results of the three type of spatial analysis- direct cell counts, cell aggregates analysis and cell neighborhood analysis between brisk vs. non-brisk TILs TME. Red boxes represent increased numbers and gray boxes–decreased numbers; pink boxes represent no change of the cell counts.

Applying our algorithms to images obtained from MxIF, we were able to identify tumor areas with T-cell infiltration within TME. Additional single cell analyses were then applied within these areas. Cellular features and likely associations were compared between brisk TIL area and non-brisk TIL (Fig 9). Different patterns in cell composition and spatial distance are clearly observed between these two types of TME micro-anatomic locations. This approach allows us to distinguish different types of TME based on their potential functional importance, by integrating multiple cellular expression features, functional profiles, and spatial information. It enables researcher to further dissect underlying key regulators in orchestrating the anti-immune response by comparing signatures between different TMEs, especially in the setting of anti-tumor treatment. Since the MxIF system can incorporate multiple cellular markers (up to 100), this analytic approach can be used to systemic assess the patterns of large amount of cell subtypes, based on different pre-specified TME of interest (e.g. CD8+PD1+ high vs. CD8+PD1+ low areas).

Tumor cell expression of HLA-1, key molecules involved in cellular antigen presentation machinery (APM), is indispensable for T-cell–dependent tumor elimination. Tumor antigens, including neoantigens, are presented to the cell surface by HLA-1 molecules [35–37] where they can be “seen” by TIL. In turn, TILs, (particularly cytotoxic T lymphocytes, CTLs), recognize these HLA-1/neoantigen complexes, induce tumor cell death, and cause tumor regression [33, 34]. In the absence of HLA-1, tumor cells are invisible to CTLs, and therefore immune to CTL destruction [24]. Tumor HLA-1 expression can be regulated by various internal or external mechanisms specific to tumor cells within TME, especially interferon-gamma. Downregulation of tumor HLA-1 expression occurs in 20% to 60% of solid malignancies [38–40]. Loss of tumor HLA-1 expression may lead to immunotherapy failure in melanoma [30, 41, 42]. Changes in HLA-1 and HLA class II molecules correlate with the clinical course of early stage melanoma [31, 32], and other malignancies [43, 44]. These changes also can predict the efficacy of immunotherapy in bladder cancer [45] and chemotherapy in ovarian cancer [46]. Our results here showed that tumor HLA-1 expression can affect tumor T cell infiltration as well as the distribution of other immune subtypes. However, these interactions are clearly heterogeneous within the TME, and tumor HLA-1 alone is not enough to predict the clinical or treatment outcomes. More interestingly, tumors with high HLA-1 expressions do not always have high TILs, indicating complex cellular regulatory network within the heterogeneous TME can impact the outcome of anti-tumor immune response. Using our algorithms, we showed that the underlying interactions between tumor and immune cells are different between sub-anatomic locations within TME, namely tumor areas with brisk vs. non-brisk TILs (Fig 9). CD3+ T cells are in more proximity with HLA-1+ tumor within the infiltrated areas than outside. Comparing to brisk TIL areas, tumor with non-brisk TIL areas contained more HLA-1 negative tumor cells and immunosuppressive cells, such as FOXP3+ T cells. These differences between different sub-anatomic TME areas supports the role of tumor HLA-1 expression in the anti-tumor immune response, however, given the functional and spatial distribution heterogeneity of immune subtypes, tumor HLA-1 expression alone is unlikely to precisely predict the outcome of anti-tumor activity. Our approach can facilitate the understanding of the underlying cell-cell interaction and regulation that favors tumor invasion vs. evasion, by analyzing and comparing features between areas of tumor growth vs. elimination. In this study, we established the concept and methodology of the systemic analytic approach to understand the TME, however, is limited by the small sample size. Applying our approaches to a larger cohort of samples in the setting of immunotherapy in future prospective study will provide invaluable insight in understanding the regulation of anti-tumor immune response in TME.

In summary, our study established a novel platform for visualization and spatial representation of cellular heterogeneity within TME, providing novel data processing algorithms necessary for understanding the cellular interplay that mediates tumor immune surveillance. This platform enables dissection of the complex TME, the first step in elucidation of the “homogeneous pattern” within the prohibitively “heterogeneous” TME, potentially allowing improved outcomes of current immunotherapies that provide durable responses in only a subset of patients.

## Supporting information

S1 Table Antibodies and their concentrations used in MxIF microscopy. Abbreviations: HLA, HLA antigen; MxIF, multiplexed immunofluorescence; PD1, programmed death protein 1.
^a^ Used as a cocktail in a 1:2 ratio.
(DOCX)

S2 Table Tumor HLA-1 expression is not associated with clinical responses to systemic immune checkpoint inhibitor treatment^a^. Abbreviations: CR, complete response; HLA-1, human leukocyte antigen 1; HLA-1 high, HLA-1 score of 3; HLA-1 low, HLA-1 score ≤2; PD, disease progression; PR, partial response; SD, stable disease.
^a^ Clinical responses (per Response Evaluation Criteria in Solid Tumors) with immunotherapy of the 28 patients shown in Fig 3B.
^b^ Overall response rate equals (CR+PR+SD)/total number of patients. ^c^ *P* = .40.
(DOCX)

S1 Fig High expression of melanoma HLA-1 correlates with high tumor-infiltrating CD8+ lymphocytes. Composite tumor microarray was performed on tissue samples obtained from 173 stage III melanoma biopsy samples. Immunohistochemistry was performed using antibody against HLA-A and CD8, both scored on a scale of 0 to 3+ (scorable HLA-A staining samples, 166; scorable HLA-A and CD8 staining samples, 164). A, Subset of 166 patients by score of tumor HLA-A expression for all samples. B, Representative tumor microarray images from 2 patients show that high HLA-1 expression on the melanoma is associated with high CD8+ TILs. C, Scoring groups of tumor-infiltrating CD8+ lymphocytes on all tumor microarray samples. Each CD8+ scoring group is shown with percentage of samples that had the HLA-A score. D, All samples with a CD8+ score of 3 and their HLA-1 expression scores. H&E indicates hematoxylin-eosin; HLA-A, HLA antigen A; TIL, tumor-infiltrating lymphocyte.
(TIF)

S2 Fig Tumor heterogeneity in melanoma TME A, B. Heterogeneous expression of melanoma HLA-1 in lymph node metastases. Formalin-fixed paraffin-embedded lymph node metastases from patients with advanced melanoma were applied to multiplexed immunofluorescence (MxIF) method. A, Heterogeneous melanoma-associated HLA-1 expression within the tumor. Top panel shows regions of interest (ROIs) from a tumor biopsy used for MxIF. Bottom panel: Two areas (black arrow) from the tumor excisional biopsy were applied to MxIF for S100B and HLA-1. vH&E are shown on the left. The percentages of tumor cells positive for HLA-1 expression are shown on the right (9.02% for area 1 and 43.70% for area 2). B, Heterogeneous HLA-1 expression in melanoma from different patients. Left panel shows ROIs selected from 2 patients’ tumor biopsies used for MxIF. Yellow arrows indicate the representative ROIs that were applied to MxIF for S100B and HLA-1; vH&E images are shown in middle panel. Tumor cells positive for HLA were 9.02% for patient 1 and 92.45% for patient 2. HLA-1 indicates HLA antigen 1; vH&E, virtual hematoxylin-eosin. C. Representative images of MxIF performed on lymph node excisional biopsy of 2 patients. Zoomed-in images of areas in yellow squares are shown on each side. HLA-1 indicates HLA antigen 1. D. CD20+PD1+ B cells in tumor microenvironment with low expression of tumor HLA antigen 1. Formalin-fixed paraffin-embedded tissue sections from lymph node metastases of patients with advanced melanoma were applied to multiplexed immunofluorescence using indicated antibodies. Zoomed-in images of areas in yellow squares are shown on each side. HLA-1 indicates HLA antigen 1.
(TIF)

S3 Fig High expression of tumor HLA-1 and high CD8+ TILs are associated with improved OS for patients with stage III melanoma. Survival outcomes are shown for 138 patients with stage III melanoma (whose biopsies were used in tumor microarray of S1 Fig). A, Patients grouped by TIL level and OS, defined as time of stage 3 diagnosis to time of death or last follow-up. B, Patients grouped by HLA-1 level and OS. C, Patients grouped by TIL and HLA-1 levels and OS. HLA-1 indicates HLA antigen 1; HLA-1 high, HLA-1 score of 3; HLA-1 low, HLA-1 score ≤2; OS, overall survival; TIL, tumor-infiltrating lymphocyte; TIL high, CD8+ score of 3; TIL low, CD8+ score ≤2.
(TIF)

S4 Fig Tumor HLA-1 expression and CD8+ TILs have no impact on PFS of 138 patients with stage III melanoma. A, TIL levels and PFS (defined as time of stage 3 diagnosis to time of progression). B, HLA-1 levels and PFS. C, Both HLA-1 and TIL levels and PFS. HLA-1 indicates HLA antigen 1; HLA-1 high, HLA-1 score of 3; HLA-1 low, HLA-1 score ≤2; PFS, progression-free survival; TIL, tumor-infiltrating lymphocyte; TIL high, CD8+ score of 3; TIL low, CD8+ score ≤2.
(TIF)

S5 Fig High tumor HLA-1 expression is associated with a tendency for better overall survival (OS) in patients with stage III melanoma who received treatments with immune checkpoint inhibitors on progression to stage IV disease. The OS of 138 stage III melanoma patients (whose biopsies were used in tumor microarray, as shown in S1 Fig) were included. Patients were grouped according to their HLA-1 and OS (defined as the time of stage 3 diagnosis to the time of death or last follow-up). A, OS in patients who did not receive any systemic immunotherapy. B, OS in patients who received systemic immunotherapy on progression to stage IV disease. HLA-1 indicates HLA antigen 1; HLA-1 high, HLA-1 score of 3; HLA-1 low, HLA-1 score ≤2; TIL high, CD8+ score of 3; TIL low, CD8+ score ≤2.
(TIF)

S1 File Supporting data set for Fig 1. (CSV)

S2 File Supporting data set for Fig 4. (CSV)

S3 File Supporting data set for Fig 7. (CSV)

S4 File Supporting data set for Fig 8. (CSV)

S1 Dataset Supporting data set. (CSV)
