# Supplementary material for: Understanding heterogeneous tumor microenvironment in metastatic melanoma
Source: PLoS One. 2019 Jun 5;14(6):e0216485. doi: 10.1371/journal.pone.0216485 (PMC6550385; doi:10.1371/journal.pone.0216485)
Supplement: S1 Table — Abbreviations: HLA, HLA antigen; MxIF, multiplexed immunofluorescence; PD1, programmed death protein 1. a Used as a cocktail in a 1:2 ratio. (DOCX) [file pone.0216485.s001.docx]

**Supporting Information Table 1**

| **Target** | **Imaging Channel** | **Antibody Clone** | **Supplier/Catalog No.** | **Concentration, ug/mL** |
| --- | --- | --- | --- | --- |
| CD11c | Cy3 | 5D11 | Leica/CD11C 563 | 5 |
| CD56 | Cy5 | MRQ-42 | Cellmarque/156R 95 | 5 |
| S6 | Cy3 | 5G10 | Cellsignaling/2217BF | 10 |
| CD34 | Cy5 | B1-3C5 | Santa Cruz/SC 19621 | 10 |
| NaKATPase | Cy3 | EP1845Y | Abcam/ab167390 | 5 |
| FOXP3 | Cy5 | 206D | Biolegend/320114 | 10 |
| CD4 | Cy3 | EPR6855 | Abcam/ab181724 | 10 |
| AE1/PCK26^a^ | Cy5 | AE1; PCK26 | AE1-ebioscience 14-9001; PCK26-Sigma C1801 | 5 |
| CD20 | Cy3 | EP459Y | Epitomics/1632-X | 5 |
| CD8 | Cy5 | C8/144B | DAKO/M7103 | 5 |
| pan_Cadherin | Cy3 | Polyclonal | Neomarkers/RB-9036-P | 10 |
| CD68 | Cy5 | KP1 | Thermo Fisher/MS-397-P1ABX | 5 |
| CD31 | Cy3 | 89C2 | Cellsignaling/3528BF | 2.5 |
| CD3 | Cy5 | F7.2.38 | DAKO/M7254 | 5 |
| S100B | Cy3 | Polyclonal | DAKO/Z0311 | 10 |
| PD1 | Cy5 | EPR4877(2) | Epitomics/6796-1 | 10 |
| Granzyme B | Cy3 | GrB-7 | DAKO/M7235 | 10 |
| Cleaved caspase 3 | Cy5 | 5A1E | Cellsignaling/9664BF | 10 |
| HLA class 1 | Cy3 | EMR8-5 | Abcam/ab70328 | 10 |
| Ki67 | Cy5 | EPR3610 | Abcam/ab196907 | 10 |
| ALDH1 | Cy5 | 44/ALDH | BD Biosciences/611195 | 10 |
