# Supplementary material for: Understanding heterogeneous tumor microenvironment in metastatic melanoma
Source: PLoS One. 2019 Jun 5;14(6):e0216485. doi: 10.1371/journal.pone.0216485 (PMC6550385; doi:10.1371/journal.pone.0216485)
Supplement: S2 Table — Abbreviations: CR, complete response; HLA-1, human leukocyte antigen 1; HLA-1 high, HLA-1 score of 3; HLA-1 low, HLA-1 score ≤2; PD, disease progression; PR, partial response; SD, stable disease. a Clinical responses (per Response Evaluation Criteria in Solid Tumors) with immunotherapy of the 28 patients shown in Fig 3B. b Overall response rate equals (CR+PR+SD)/total number of patients. c P = .40. (DOCX) [file pone.0216485.s002.docx]

**Supporting Information Table 2**

| **Clinical Response^a^** | **CR** | **PR** | **SD** | **PD** | **Overall Response Rate, %^b,c^** |
| --- | --- | --- | --- | --- | --- |
| HLA-1 high | 1 | 6 | 3 | 10 | 50 |
| HLA-1 low | 2 | 0 | 0 | 6 | 25 |
