# Supplementary figures and images for: Understanding heterogeneous tumor microenvironment in metastatic melanoma
Source: PLoS One. 2019 Jun 5;14(6):e0216485. doi: 10.1371/journal.pone.0216485 (PMC6550385; doi:10.1371/journal.pone.0216485)

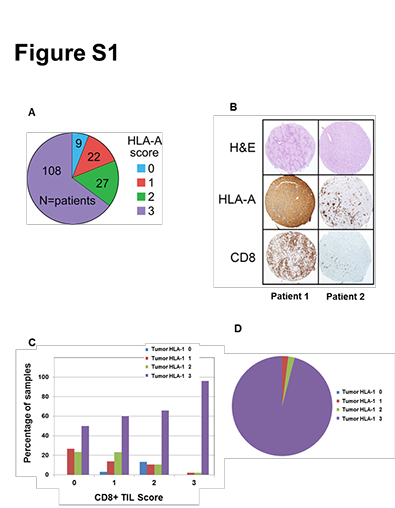

Supplement: S1 Fig — Composite tumor microarray was performed on tissue samples obtained from 173 stage III melanoma biopsy samples. Immunohistochemistry was performed using antibody against HLA-A and CD8, both scored on a scale of 0 to 3+ (scorable HLA-A staining samples, 166; scorable HLA-A and CD8 staining samples, 164). A, Subset of 166 patients by score of tumor HLA-A expression for all samples. B, Representative tumor microarray images from 2 patients show that high HLA-1 expression on the melanoma is associated with high CD8+ TILs. C, Scoring groups of tumor-infiltrating CD8+ lymphocytes on all tumor microarray samples. Each CD8+ scoring group is shown with percentage of samples that had the HLA-A score. D, All samples with a CD8+ score of 3 and their HLA-1 expression scores. H&E indicates hematoxylin-eosin; HLA-A, HLA antigen A; TIL, tumor-infiltrating lymphocyte. (TIF) [file pone.0216485.s003.tif]

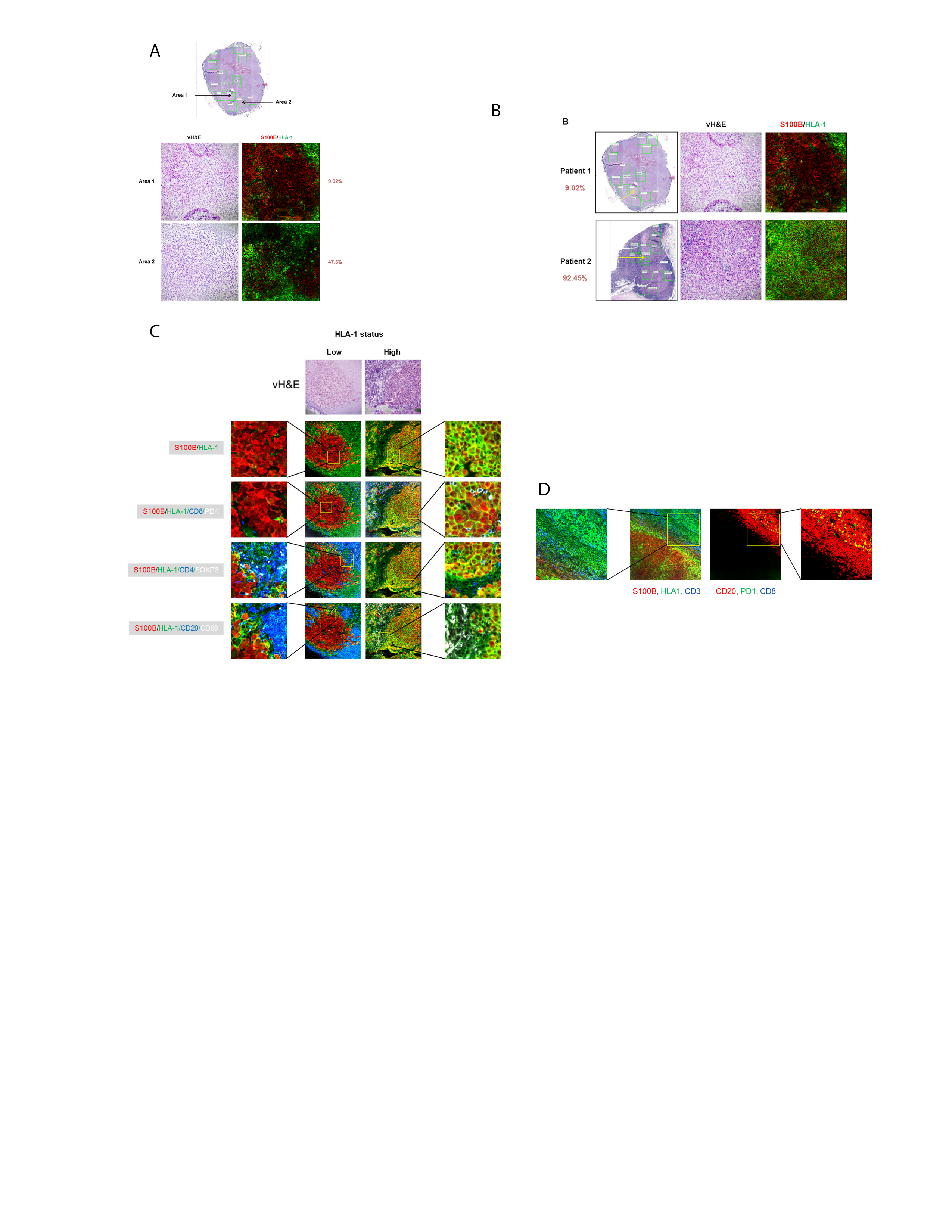

Supplement: S2 Fig — Tumor heterogeneity in melanoma TME A, B. Heterogeneous expression of melanoma HLA-1 in lymph node metastases. Formalin-fixed paraffin-embedded lymph node metastases from patients with advanced melanoma were applied to multiplexed immunofluorescence (MxIF) method. A, Heterogeneous melanoma-associated HLA-1 expression within the tumor. Top panel shows regions of interest (ROIs) from a tumor biopsy used for MxIF. Bottom panel: Two areas (black arrow) from the tumor excisional biopsy were applied to MxIF for S100B and HLA-1. vH&E are shown on the left. The percentages of tumor cells positive for HLA-1 expression are shown on the right (9.02% for area 1 and 43.70% for area 2). B, Heterogeneous HLA-1 expression in melanoma from different patients. Left panel shows ROIs selected from 2 patients’ tumor biopsies used for MxIF. Yellow arrows indicate the representative ROIs that were applied to MxIF for S100B and HLA-1; vH&E images are shown in middle panel. Tumor cells positive for HLA were 9.02% for patient 1 and 92.45% for patient 2. HLA-1 indicates HLA antigen 1; vH&E, virtual hematoxylin-eosin. C. Representative images of MxIF performed on lymph node excisional biopsy of 2 patients. Zoomed-in images of areas in yellow squares are shown on each side. HLA-1 indicates HLA antigen 1. D. CD20+PD1+ B cells in tumor microenvironment with low expression of tumor HLA antigen 1. Formalin-fixed paraffin-embedded tissue sections from lymph node metastases of patients with advanced melanoma were applied to multiplexed immunofluorescence using indicated antibodies. Zoomed-in images of areas in yellow squares are shown on each side. HLA-1 indicates HLA antigen 1. (TIF) [file pone.0216485.s004.tif]

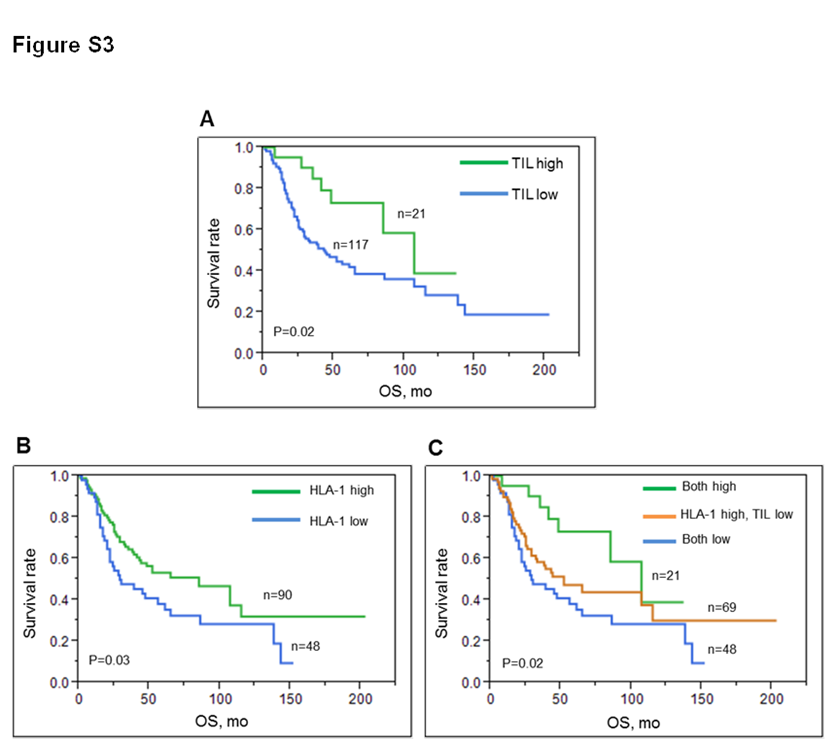

Supplement: S3 Fig — Survival outcomes are shown for 138 patients with stage III melanoma (whose biopsies were used in tumor microarray of S1 Fig). A, Patients grouped by TIL level and OS, defined as time of stage 3 diagnosis to time of death or last follow-up. B, Patients grouped by HLA-1 level and OS. C, Patients grouped by TIL and HLA-1 levels and OS. HLA-1 indicates HLA antigen 1; HLA-1 high, HLA-1 score of 3; HLA-1 low, HLA-1 score ≤2; OS, overall survival; TIL, tumor-infiltrating lymphocyte; TIL high, CD8+ score of 3; TIL low, CD8+ score ≤2. (TIF) [file pone.0216485.s005.tif]

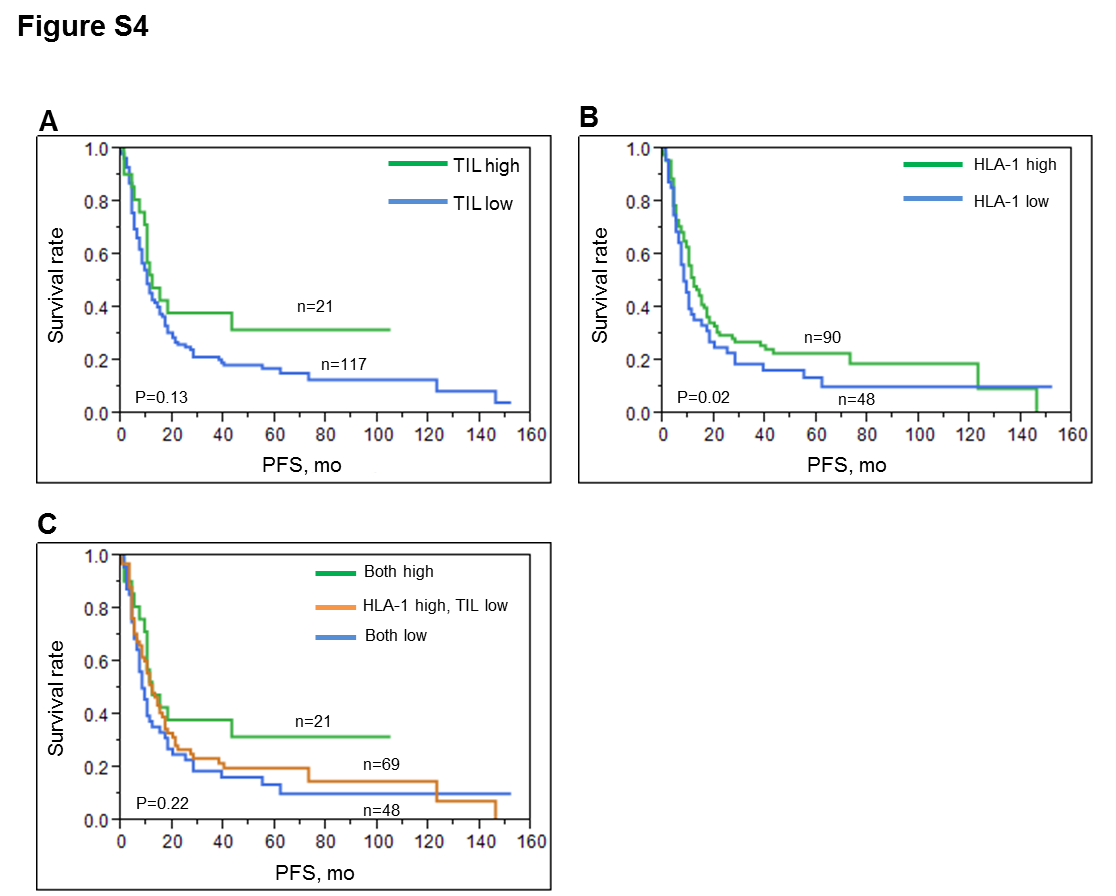

Supplement: S4 Fig — A, TIL levels and PFS (defined as time of stage 3 diagnosis to time of progression). B, HLA-1 levels and PFS. C, Both HLA-1 and TIL levels and PFS. HLA-1 indicates HLA antigen 1; HLA-1 high, HLA-1 score of 3; HLA-1 low, HLA-1 score ≤2; PFS, progression-free survival; TIL, tumor-infiltrating lymphocyte; TIL high, CD8+ score of 3; TIL low, CD8+ score ≤2. (TIF) [file pone.0216485.s006.tif]

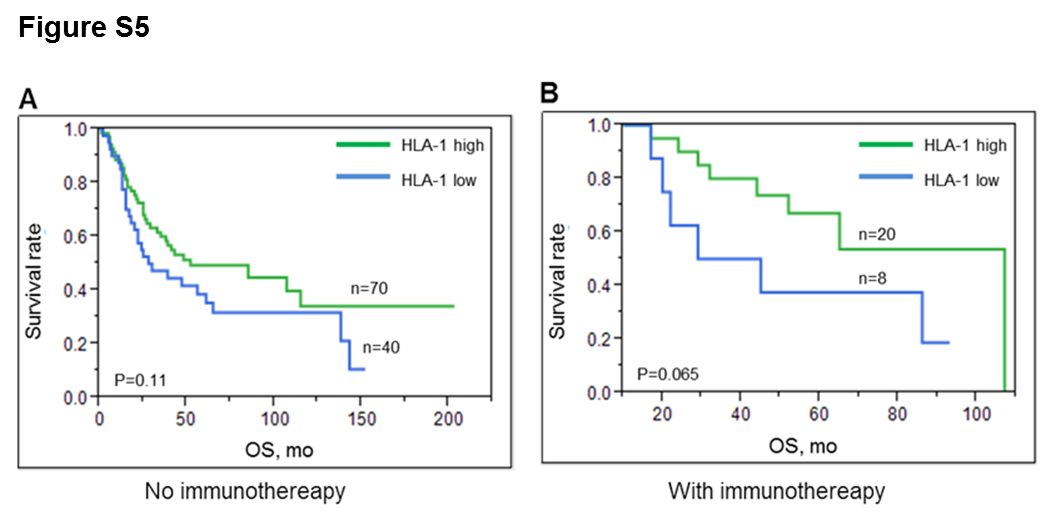

Supplement: S5 Fig — The OS of 138 stage III melanoma patients (whose biopsies were used in tumor microarray, as shown in S1 Fig) were included. Patients were grouped according to their HLA-1 and OS (defined as the time of stage 3 diagnosis to the time of death or last follow-up). A, OS in patients who did not receive any systemic immunotherapy. B, OS in patients who received systemic immunotherapy on progression to stage IV disease. HLA-1 indicates HLA antigen 1; HLA-1 high, HLA-1 score of 3; HLA-1 low, HLA-1 score ≤2; TIL high, CD8+ score of 3; TIL low, CD8+ score ≤2. (TIF) [file pone.0216485.s007.tif]
